# ^18^F-DOPA PET/MRI With Carbidopa for the Diagnosis of Hyperinsulinemic Hypoglycemia in an Adolescent Patient

**DOI:** 10.1210/jcemcr/luae153

**Published:** 2024-08-21

**Authors:** Carine Anka, Maria Rosana Ponisio, Patrick A Dillon, Chelsea Schmitt, Tyler J Fraum, Ana María Arbeláez

**Affiliations:** Department of Pediatrics, Washington University, St Louis, MO 63110, USA; Department of Radiology, Washington University, St Louis, MO 63110, USA; Department of Surgery, Washington University, St Louis, MO 63110, USA; Department of Radiology, Washington University, St Louis, MO 63110, USA; Department of Radiology, Washington University, St Louis, MO 63110, USA; Department of Pediatrics, Washington University, St Louis, MO 63110, USA

**Keywords:** hyperinsulinism, insulinoma, children, ^18^F-DOPA, PET/MRI, carbidopa

## Abstract

Hyperinsulinism due to focal or diffuse pancreatic lesions causing recurrent episodes of hypoglycemia is rare in mid-childhood. There is no consensus on the gold-standard imaging method to diagnose focal insulin-producing lesions beyond infancy. A 14-year-old boy with a complex medical history and refractory epilepsy, presented with blood glucose (BG) of 52 mg/dL (2.9 mmol/L) (normal reference range: 70-100 mg/dL [3.9-5.6 mmol/L]) and increased seizure frequency. He failed a fast within 4 hours, with BG of 48 mg/dL (2.7 mmol/L) and insulin level of 4.6 µIU/mL (24.6 pmol/L) (diagnostic at the time of hypoglycemia >1.25 μU/mL [8.7 pmol/L]). Conventional imaging studies showed no pancreatic lesion. Fluorine-18-L-dihydroxyphenylalanine positron emission tomography/magnetic resonance imaging (^18^F-DOPA-PET/MRI) scan premedicated with carbidopa demonstrated intense focal ^18^F-DOPA uptake in the distal pancreatic tail. He underwent distal pancreatectomy. Histopathology showed focal pancreatic islet cell hyperplasia, with more than 90% of the neuroendocrine islet cells being positive for chromogranin and synaptophysin, with no loss of p57 staining. Genetic studies were negative for mutations in *ABCC8*, *KCNJ11*, *GCK*, or *GLUD1* genes, multiple endocrine neoplasia (MEN) type 1, and Beckwith-Wiedemann syndrome. BG normalized after surgery. Seizure frequency improved. This case highlights the utility of ^18^F-DOPA PET/MRI imaging in diagnosing focal hyperinsulinism beyond infancy.

## Introduction

Hyperinsulinemic hypoglycemia (HH) can result from a variety of transient and persistent factors affecting children and adults. Persistent hyperinsulinism in infants and young children is typically due to defects in distinct genes (*ABCC8*, *KCNJ11*, *GLUD1*, *GCK*, *HADH*, *SLC16A1*, *HNF4A*, *HNF1A*, and *UCP2*) that are responsible for regulating insulin release and can result in focal or diffuse histopathological subtypes [1, 2]. In adults and older children, severe HH is often due to an insulinoma, an insulin-secreting tumor of the pancreas [2]. Clinical diagnosis of hyperinsulinism is confirmed by inappropriately elevated insulin, elevated C-peptide, and suppressed β-hydroxybutyrate levels at the time of hypoglycemia, with resolution of symptoms after glucose administration, satisfying the diagnostic criteria known as the Whipple triad. Unrecognized hypoglycemia can result in seizures and long-term neurodevelopmental problems [2]. Therefore, prompt detection of potential pancreatic focal lesions resulting in hyperinsulinism in children or adults is critical, as resection can cure the disease. Ultrasound, contrast-enhanced computed tomography (CT) and contrast-enhanced magnetic resonance imaging (MRI) are frequently used to detect pancreatic abnormalities. Despite being widely available and easily accessible, the aforementioned methods are associated with variable sensitivity and limitations. The small size of some of these lesions and the suboptimal sensitivity and specificity of these imaging methods make localization challenging [2]. Over the last 3 decades, fluorine-18-L-dihydroxyphenylalanine positron emission tomography (^18^F-DOPA PET) has played a crucial role in diagnosing and subsequent targeted treatment strategies of congenital hyperinsulinism (CHI) in infants and young children. In the systematic review and meta-analysis by Blomberg [3], ^18^F-DOPA PET outperformed pancreatic venous sampling (sensitivity 87%, specificity 73%) and hepatic venous sampling (sensitivity 71%, specificity 69%) when it came to distinguishing between focal and diffuse CHI in infants and children [3]. Specifically, ^18^F-DOPA PET exhibited a sensitivity of 75% and a specificity of 100%. Likewise, in insulinomas, preoperative localization is crucial to facilitate pancreas-preserving surgery. However, these rare pancreatic islet cell tumors are usually sporadic, solitary, and less than 2 cm in diameter, making their detection challenging by conventional imaging techniques (with varying sensitivity) [4]. A few studies have described the clinical value of ^18^F-DOPA in detecting insulinomas. Since ^18^F-DOPA can be taken up by the whole pancreatic gland, administering carbidopa before imaging can be a helpful adjunct in adults since it lowers physiologic pancreatic uptake, increasing the conspicuity of focal lesions [5]. Others have used ^18^F-DOPA-PET/CT with contrast. However, PET/CT comes with the risk of additional ionizing radiation from CT localization, which is of particular concern in the pediatric population. Whole-body PET/MR has been shown to have a 73% radiation dose reduction compared to whole-body PET/CT in pediatric patients [6]. This paper presents a unique case of a child with hyperinsulinism in which ^18^F-DOPA PET/MRI in combination with oral carbidopa allowed for successful localization of focal β-cell hyperplasia, which was subsequently confirmed by histology, and the patient's hypoglycemia resolved after resection.

## Case Presentation

A 14-year-old boy with quadriplegic cerebral palsy, bilateral perisylvian syndrome, schizencephaly, refractory epilepsy, ventilator-dependent chronic respiratory failure, and a gastrostomy tube presented to the emergency department for increased frequency of baseline seizure activity. His initial workup in the emergency department was notable for a blood glucose (BG) of 52 mg/dL (2.9 mmol/L), (normal reference range, 70-100 mg/dL [3.9-5.6 mmol/L]), initially attributed to a recently missed feed. Consequently, he received a 2-mL/kg 10% dextrose bolus and started on dextrose-containing fluids. He was admitted for further evaluation of his increased seizure activity.

## Diagnostic Assessment

**Figure 1.**
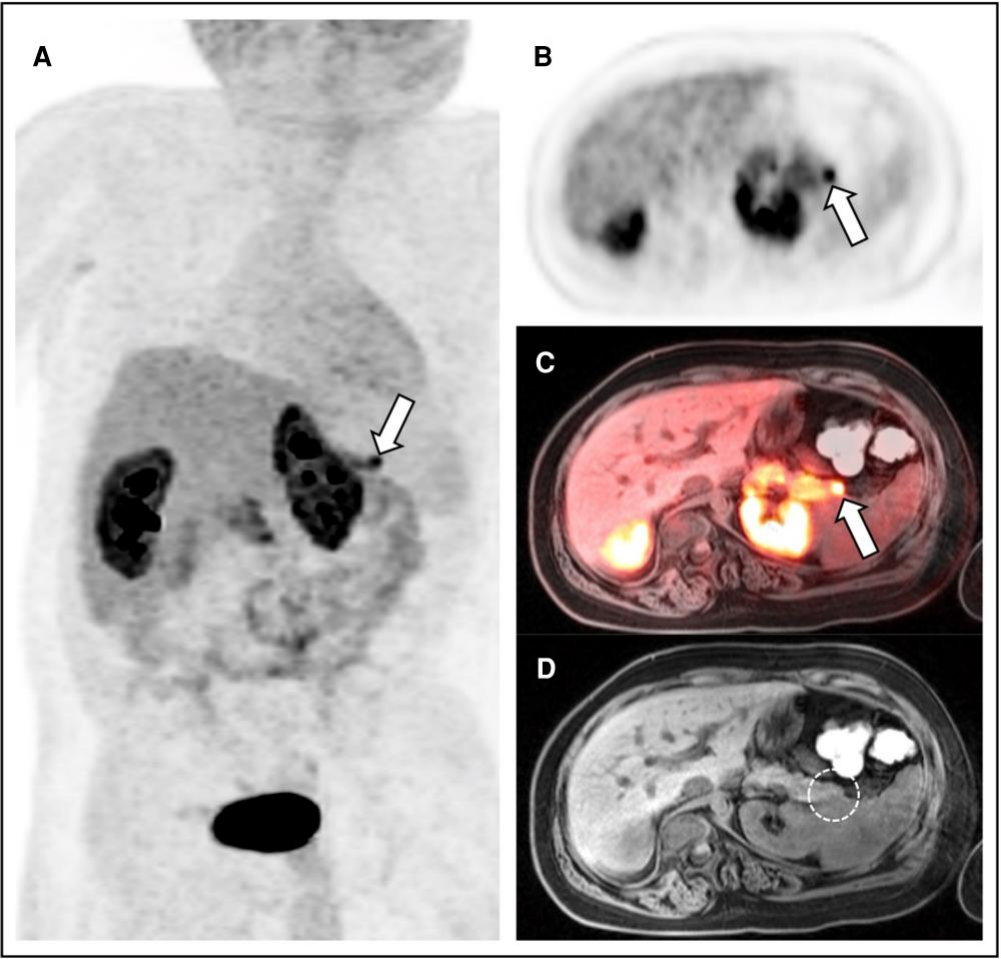
Coronal maximum-intensity-projection (MIP) A, ^18^F-DOPA positron emission tomography (PET); B, axial ^18^F-DOPA PET; C, fused axial ^18^F-DOPA PET/magnetic resonance imaging (MRI); and D, axial noncontrast T1-weighted MRI show a focal intensely tracer-avid lesion (arrows) localizing to the tip of the pancreatic tail but without an anatomic correlate (circle).

At 4 hours of a diagnostic fast, the patient's serum BG was 48 mg/dL (2.7 mmol/L), insulin level was 4.6 µIU/mL (24.6 pmol/L) (diagnostic at time of hypoglycemia >1.25 μU/mL [8.7 pmol/L]) [7], elevated C-peptide was 6.9 ng/mL (2.28 nmol/L) (diagnostic at time of hypoglycemia >0.5 ng/mL [>0.17 nmol/L]) [7], suppressed β-hydroxybutyrate was 0.1 mmol/L (1 mg/dL) (diagnostic at time of hypoglycemia <1.8 mmol/L) [7], normal ammonia was 48 µmol/L (82 µg/dL) (normal range 9-54 μmol/L), free fatty acids were 0.24 mmol/L (diagnostic at time of hypoglycemia <1.7 mmol/L) [7], and low cortisol was 2.5 µg/dL (69 nmol/L) (normal range, 6.24-18.0 μg/dL [172.2-496.8 nmol/L]). At the time of low BG, he did not appear distressed. No observed clinical seizure or neurogenic or neuroglycopenic symptoms were present. His heart rate was 52 to 62 beats per minute during the fast. A 1-mcg low-dose cosyntropin stimulation induced a peak cortisol response of 21.7 µg/dL (598.7 nmol/L), ruling out adrenal insufficiency. Toxicology testing for oral antidiabetic drugs was negative. The acylcarnitine profile was normal, ruling out fatty acid oxidation disorders. The patient's liver and renal function tests were unremarkable and ruled out liver or renal failure. His electrolytes including calcium and phosphorus levels were normal, and his family history was negative for multiple endocrine neoplasia (MEN) or other hypoglycemic disorders. Genetic studies did not identify known mutations in the *ABCC8*, *KCNJ11*, *GCK*, or *GLUD1* genes. Similarly, genetic assessments for MEN type 1, and methylation test for Beckwith-Wiedemann Syndrome, were negative. Chromosomal microarray showed no genomic imbalance or regions of homozygosity. A whole-exome sequencing was ordered but the mother declined consent. CT of the abdomen/pelvis with contrast and MRI of the abdomen without contrast failed to demonstrate a focal pancreatic lesion. Given the lack of diagnosis with conventional imaging, ^18^F-DOPA PET/MRI was obtained. The Saint Louis Children's Ethics Committee and the Radioactive Drug Research Committee approved this patient's compassionate use of ^18^F-DOPA. The Washington University Human Research Protection Office and the US Food and Drug Administration gave an emergency exemption to perform an ^18^F-DOPA PET/MRI on this child. The patient's guardian signed written informed consent approved by the Human Research Protection Office. Two hours before imaging, the patient was premedicated with 100 mcg of carbidopa orally once. Subsequently, an intravenous injection of 5.3 mCi of ^18^F-DOPA was administered. The uptake time from the injection of ^18^F-DOPA to the start of abdominal emission imaging was 14 minutes. A simultaneous abdominal MRI and PET dynamic acquisition was performed using a 3-T Siemens Biograph mMR PET/MRI system that lasted 60 minutes. In addition, whole-body PET images were obtained to exclude the presence of extra-abdominal disease sites. The resulting images (Fig. 1) demonstrated a 1.1 cm focus of intense ^18^F-DOPA uptake in the distal pancreatic tail, contrasting with the uptake observed in the rest of the pancreas. No discernible corresponding morphological abnormality was identified on the concurrent noncontrast MRI. The findings indicated the presence of a focal neuroendocrine tumor in the pancreatic tail, which strongly supported the possibility of an insulinoma given the clinical context.

## Treatment

After reviewing the patient's clinical course and imaging findings, the patient underwent an open distal pancreatectomy with splenic preservation. The specimen measured 5.5 × 3.0 × 2.0 cm and weighed 30 g. A microscopic examination of the pancreatic tissue revealed the presence of prominent islets containing proliferations of benign neuroendocrine cells intermingled with normal-appearing acinar cells, with no obvious nucleomegaly. Immunohistochemical stains of the lesion for chromogranin and synaptophysin were positive in more than 90% of the cells of the neuroendocrine islets, consistent with β-cell hyperplasia. There was no loss of p57 staining.

## Outcome and Follow-up

After surgery, the patient’s BG normalized and seizure frequency decreased, permitting tapering off of several antiepileptic drugs. Two years after surgery, the patient's BG remained normal when checked at home and during subsequent hospitalizations for respiratory issues.

## Discussion

Persistent endogenous hyperinsulinism presenting outside the neonatal period is a rare entity. It can result from an insulinoma, which is a functional neuroendocrine insulin-secreting tumor of the pancreas that is usually small and sporadic but can also be found in patients with MEN 1 syndrome [8]. They cause inappropriate, unregulated insulin secretion that leads to increased glucose uptake by insulin-sensitive cells and simultaneous inhibition of glycogenolysis, gluconeogenesis, lipolysis, and ketogenesis, resulting in recurrent, severe hypoglycemia, which if unrecognized can potentially cause brain injury or death. The diagnosis of endogenous hyperinsulinemia relies on both the clinical presentation and a specific biochemical profile that demonstrates detectable serum insulin, detectable C-peptide with suppressed ketones, and suppressed free fatty acids in the setting of low plasma glucose [8]. The median duration of symptoms before diagnosis is around 1.5 years [9], although some patients may have been symptomatic for decades before the diagnosis is made. However, like in this case, it is estimated that about 20% of patients with insulinoma are misdiagnosed with a neurologic, psychiatric, or seizure disorder before the insulinoma is recognized [10]. After biochemical diagnosis, precise preoperative lesion localization of the source of HH is crucial for guided surgical resection with minimal morbidity and preservation of pancreatic function [11]. However, lesion localization can be challenging, given the suboptimal sensitivity of conventional diagnostic modalities. For instance, the sensitivity of CT is reported at 64%, while that of MRI is 75% [4]. Endoscopic ultrasound and angiography with intra-arterial calcium stimulation and venous sampling are invasive and highly operator dependent, with widely varying sensitivities of 65% to 94% and 63% to 94%, respectively [12]. Using ^68^Ga-labeled somatostatin analogues has been widely accepted for imaging neuroendocrine tumors and other somatostatin-positive tumors, including meningiomas [13]. Nevertheless, the sensitivity for insulinomas is variable, which may be partly due to the varying expression of somatostatin receptors in benign and malignant insulinomas [11]. Radiolabeled glucagon-like peptide 1 analogues have shown promising results as imaging agents; however, they are not available in most imaging centers worldwide and should be used in the setting of clinical trials only [1].

More recently, ^18^F-DOPA PET has emerged as a valuable radiopharmaceutical for imaging catecholamine-secreting tumors [14]. This imaging technique can distinguish focal from diffuse pancreatic cell hyperplasia, enhancing its diagnostic utility in these complex patients. Pancreatic β cells can take up amine precursors, like L-DOPA, and convert them into dopamine via L-DOPA decarboxylase activity. Radiolabeled L-DOPA (ie,^18^F-DOPA) is transported across the β-cell membrane by an amino acid transporter, after which it is decarboxylated into ^18^F-fluoro-dopamine and stored in vesicles [14]. Carbidopa inhibits peripheral amino acid decarboxylase, with a greater effect on background pancreatic parenchymal activity. Thus, this adjuvant intervention improves the conspicuity of focal lesions by increasing the ratio of ^18^F-DOPA uptake in a focal lesion compared to background pancreatic parenchyma [5]. Though some have employed 18F-DOPA PET/CT with the administration of intravenous contrast and early acquisition time to improve detection [15], PET/CT comes with the risk of additional ionizing radiation from CT localization, which is of particular concern in the pediatric population like this case.

PET/CT is widely available with established imaging protocols and evidence of proven indication. On the other hand, PET/MRI offers superior soft tissue contrast and more accurate coregistration of anatomic and PET images due to simultaneous acquisition, facilitating anatomic localization of pancreatic lesions. Furthermore, PET/MRI affords various motion correction opportunities and reduces exposure to ionizing radiation compared to PET/CT [5]. This is particularly important when imaging pediatric patients or patients requiring multiple scans over time. However, PET/MRI continues to be of limited availability, with protocols and indications still in development and the need for technologists knowledgeable both in PET and MRI [13]. At institutions with access to this scanner and appropriately trained technologists, PET/MRI likely represents an ideal imaging modality for localizing insulinomas.

In summary, our case represents how ^18^F-DOPA PET/MR imaging with carbidopa premedication in a child represents a promising advancement in the diagnosis of focal hyperinsulinism, providing comprehensive and precise insights into this complex condition through multimodal imaging techniques. This combined approach enhances diagnostic accuracy and aids in tailoring effective treatment strategies.

## Learning Points

- Hyperinsulinemic Hypoglycemia is a rare entity in mid-childhood, thereby conferring a degree of complexity on its diagnosis.
- The integration of ^18^F-DOPA PET/MRI imaging is essential in the diagnostic process, treatment strategizing, and, ultimately, the prognostic assessment of hyperinsulinism.
- Premedication with carbidopa facilitates localization of pancreatic lesions on ^18^F-DOPA PET/MRI.

## Data Availability

Original data generated and analyzed for this case report are included in this published article.

## Abbreviations

^18^F-DOPA-PET/MRI: fluorine-18-L-dihydroxyphenylalanine positron emission tomography/magnetic resonance imaging
PET/CT: positron emission tomography/computed tomography
BG: blood glucose
CHI: congenital hyperinsulinism
HH: hyperinsulinemic hypoglycemia
MEN: multiple endocrine neoplasia
